# Supplementary material for: Loss of ARID1A accelerates prostate tumourigenesis with a proliferative collagen-poor phenotype through co-operation with AP1 subunit cFos
Source: Br J Cancer. 2025 Jan 30;132(6):502–12. doi: 10.1038/s41416-025-02944-3 (PMC11920240; doi:10.1038/s41416-025-02944-3)
Supplement: Supplementary file 1 — Supplementary Figure and Tables [file 41416_2025_2944_MOESM1_ESM.docx]

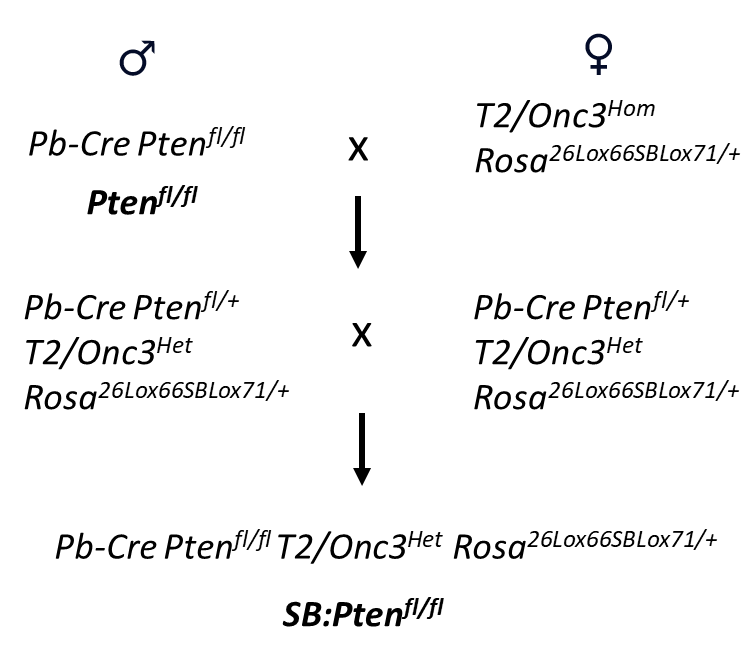


**Supplementary Figure 1**: Breeding schematic to produce *Probasin Cre Pten^fl/fl^* line with Sleeping Beauty system


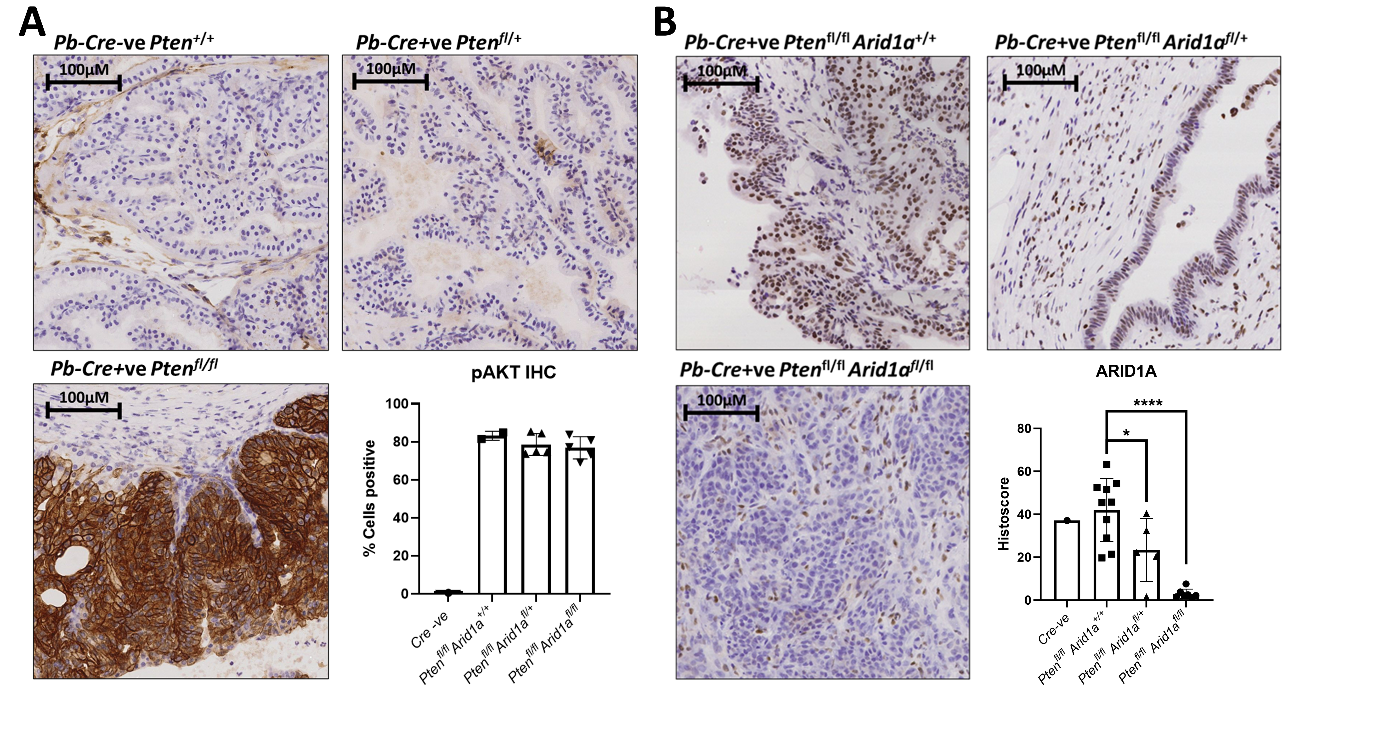


**Supplementary Figure 2: Immunohistochemistry for phospho-serine 474 AKT and ARID1A. A.** Representative staining of prostate stained for phospho-serine 474 AKT and quantification of stain-positive cells . *Pb-Cre:Pten^+/+^* shown at 6 months timepoint, *Pb-Cre:Pten^fl/+^* shown at 6 month timepoint, *Pb-Cre:Pten^fl/fl^* shown at 9 month clinical endpoint. Quantification of pAKT IHC in Cre-ve compared to *Pb-Cre:Pten^fl/fl^* with various *Arid1a* status shows ARID1A loss does not impact pAKT levels. Each data point is an individual mouse, error bars show SEM. **B.** Representative staining of endpoint prostate tumour stained for ARID1A and quantification of histoscore in indicated genotypes. Each data point represents an individual mouse, error bars show SEM.*P=0.0373, ****P<0.0001 ANOVA with Tukey’s analysis.


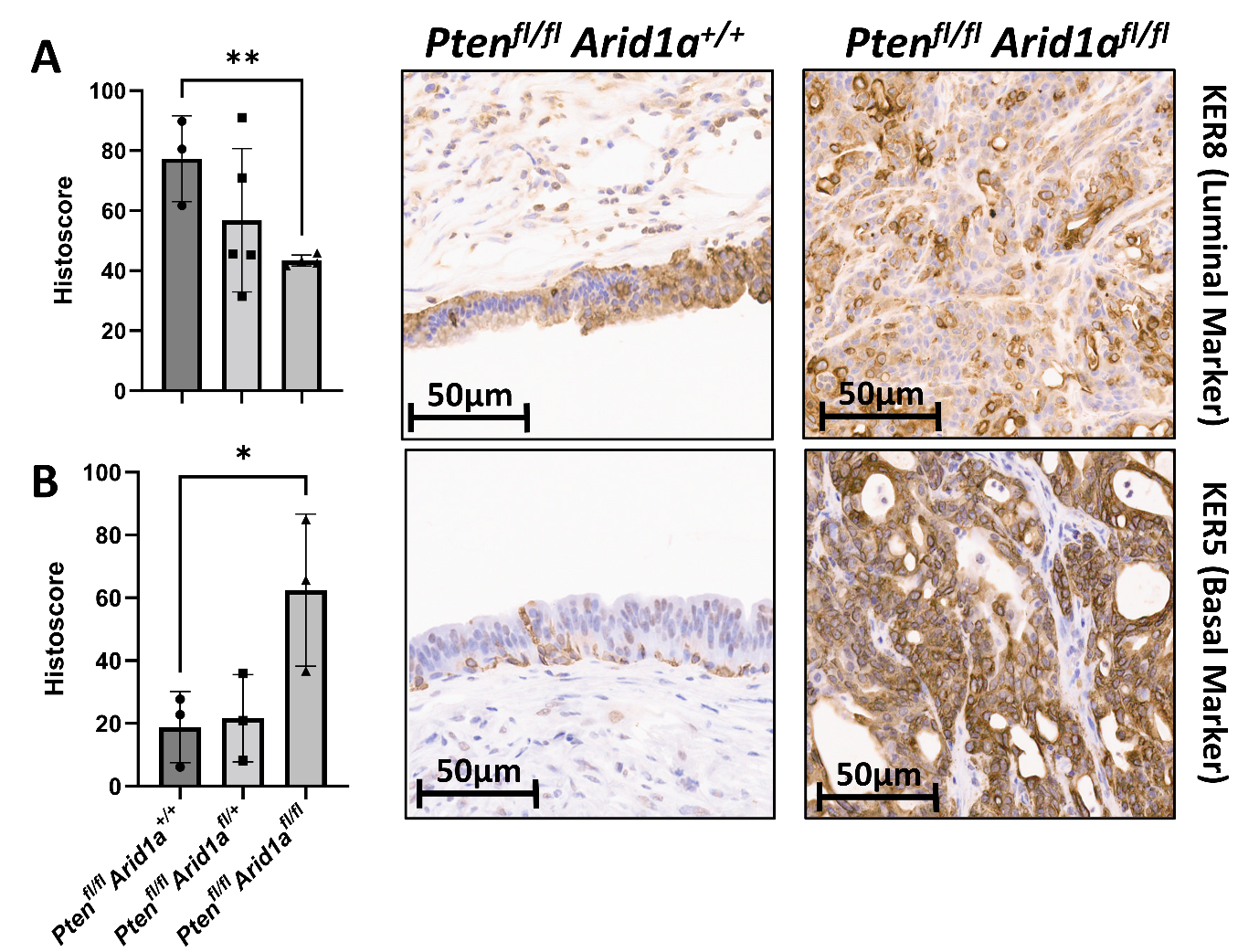


**Supplementary Figure 3: IHC for basal vs luminal markers following loss of ARID1A. A.** Representative staining of Keratin 8 in epithelial compartment of indicated genotypes. Quantification of histoscore in indicated genotype, **P=0.0047 Keratin 8; tested by ANOVA with Tukey’s post hoc analysis. Each data point is an individual tumour. **B** Representative staining of Keratin 5 in the indicated genotypes. Quantification of histoscore in epithelial compartment of indicated genotype, *P=0.048 Keratin 5; tested by ANOVA with Tukey’s post hoc analysis.

**
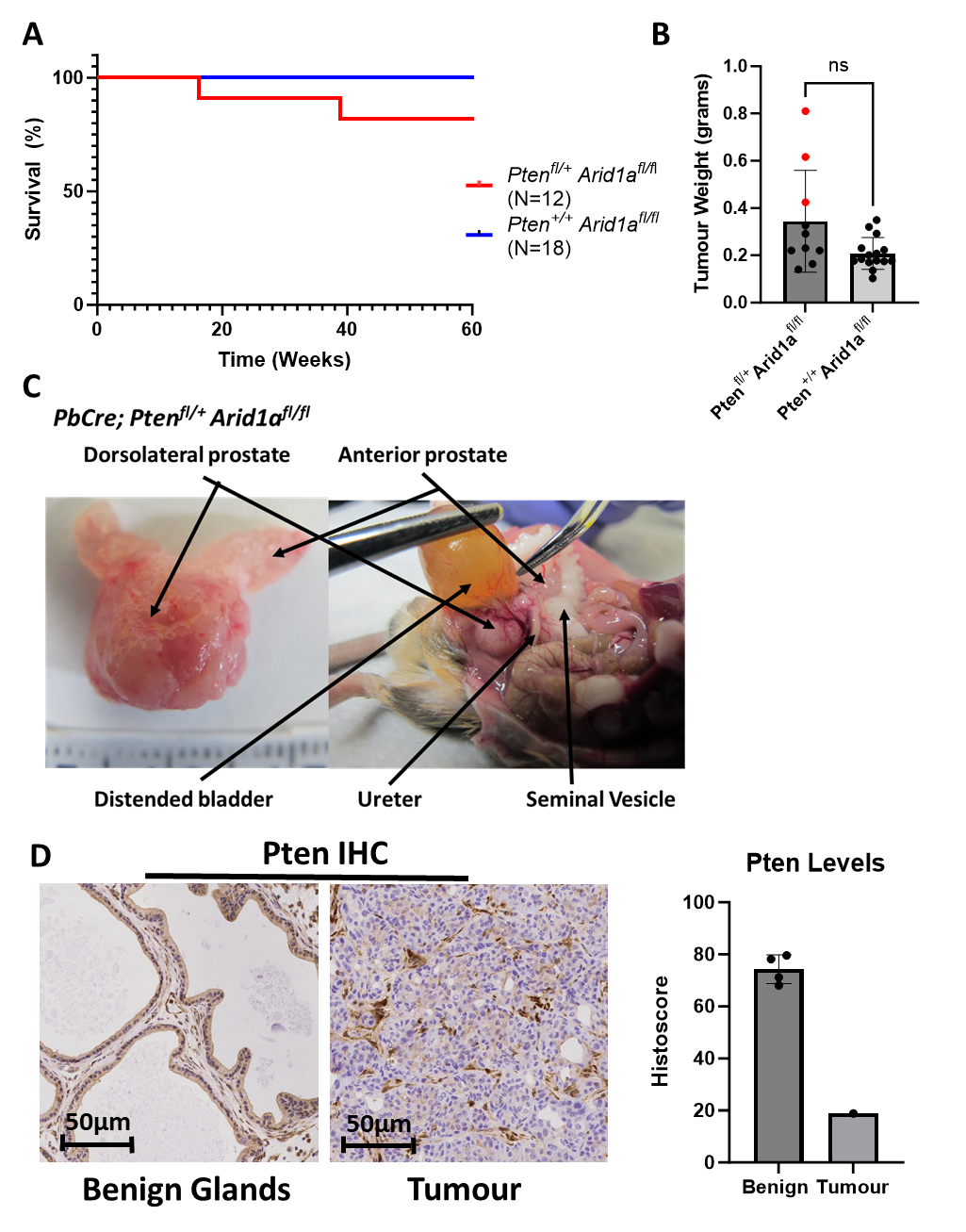
**

**Supplementary Figure 4: *Pten* loss is a prerequisite for *Arid1a* loss mediated tumorigenesis A.** Kaplan–Meier (log-rank) curve demonstrating survival of *Pb-Cre;Pten^fl/+^Arid1a^fl/fl^* (n=17) and *Pb-Cre;Pten^+/+^ Arid1a^fl/fl^* (n=18) male mice, not significant; log-rank (Mantel-Cox) test. **B.** Weight in grams of prostate/tumours harvested from male mice taken at clinical or ageing endpoint at 60 weeks of *Pb-Cre;Pten^fl/+^Arid1a^fl/fl^* (n=12 and *Pb-Cre;Pten^+/+^ Arid1a^fl/fl^* (n=18), not significant P=0.083, Mann-Whitney. Each data point represents and individual mouse, those in red reached clinical endpoint due to tumour burden. **C.** *Ex vivo* and *in situ* prostate tumour of *Pb-Cre;Pten^fl/+^Arid1a^fl/fl^* genotype with spontaneous development of dorsolateral tumour. with key structures labelled. **D.** Staining of endpoint dorsolateral prostate tumour of a *Pb-Cre;Pten^fl/+^Arid1a^fl/fl^* mouse for PTEN and ARID1A respectively with key structures labelled.


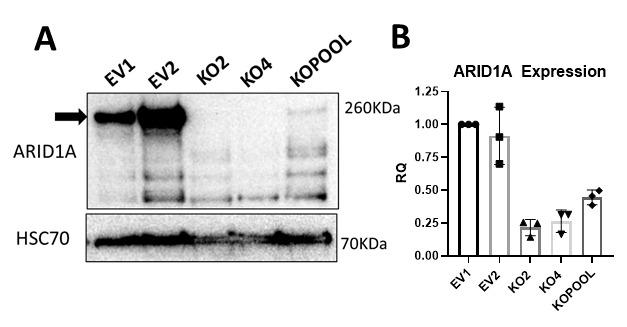


**Supplementary Figure 5: Validation of CRISPR-Cas9 Knockout of ARID1A in DU145 cells**

**A.** Immunoblotting of protein from DU145 empty vector (EV) 1 and 2 clones, and ARID1A knockout (KO) clones 2, 4, and pool for ARID1A (Molecular weight = 260 kDa). HSC70 was used as loading control. Representative blot of three experimental replicates. **B.** Quantitative RT-PCR comparing relative quantification (RQ) of *ARID1A* expression in DU145 EV clones compared to *ARID1A* KO clones. Each point represents an experimental replicate each made up of three technical replicates, error bars showing SEM.

**
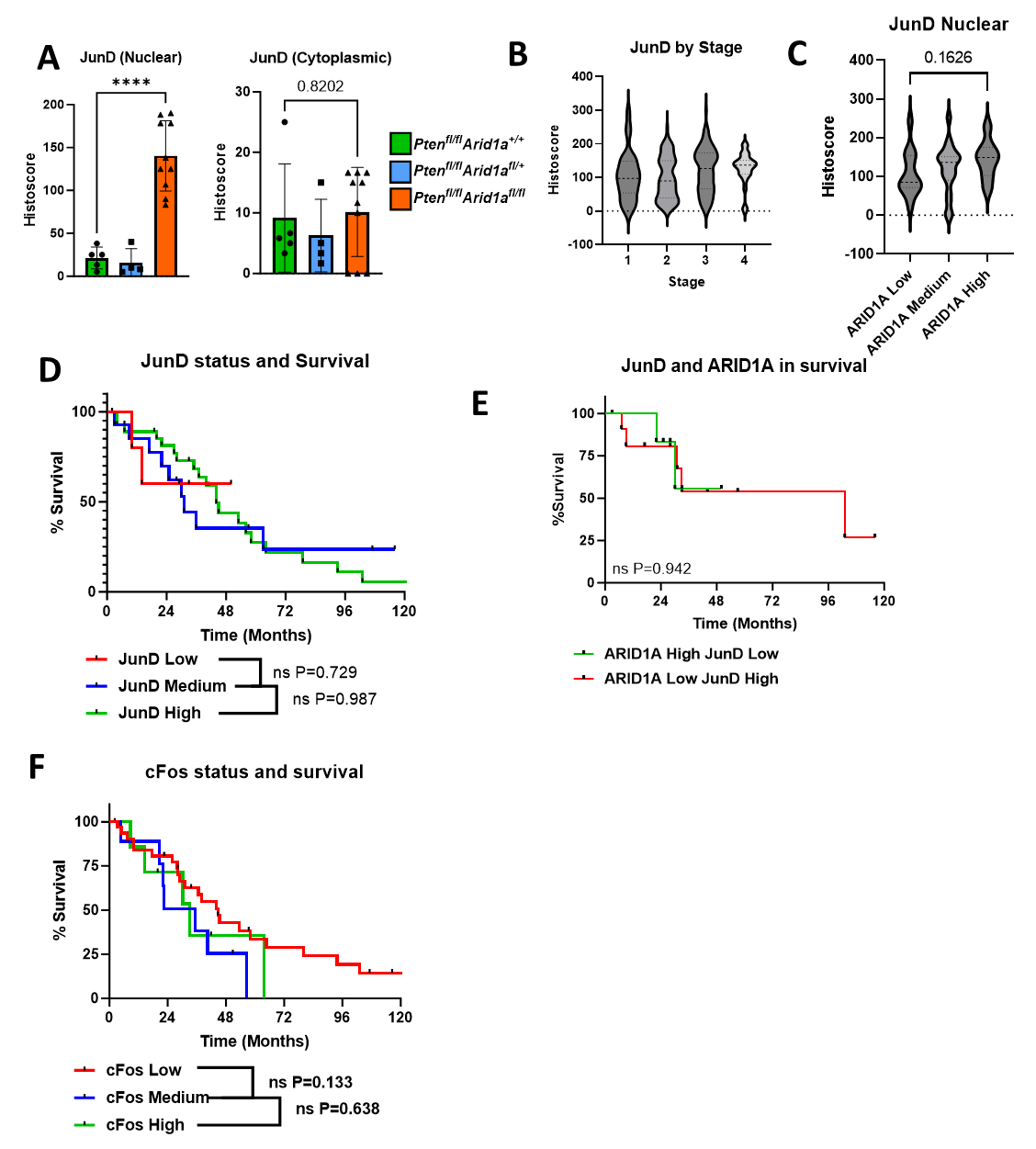
**

**Supplementary Figure 6: A. JunD status does not predict prostate cancer survival** Immunohistochemistry of indicated mouse tissue (as in Figure 5) stained and scored for nuclear and cytoplasmic positivity of JunD ****P<0.0001. Each data point is an individual mouse. **B.** Histoscore for nuclear JunD staining of human tissue microarray by stage of prostate cancer (same as in Figure 1 Iand 5). **C.** JunD positivity when compared to ARID1A status from human prostate cancer tissue microarray. Not significant, P=0.1626; ANOVA with Tukey’s analysis. **D.** Kaplan–Meier (log-rank) curve demonstrating survival of patient cohorts with different levels of JunD. JunD Low (N=5); JunD Medium (N=14); JunD High (N=28). Not significant; log-rank (Mantel-Cox) test. **E.** Kaplan–Meier (log-rank) curve demonstrating survival of patient cohorts comparing ARID1A low JunD high (N=12) vs ARID1A high JunD low (N=6). Not significant; log-rank (Mantel-Cox) test. **F.** Kaplan–Meier (log-rank) curve demonstrating survival of patient cohorts with different levels of cFos stained in the TMA. cFos Low (N=31), cFos Medium (N=10), cFos High (N=7). Not significant; log-rank (Mantel-Cox) test.


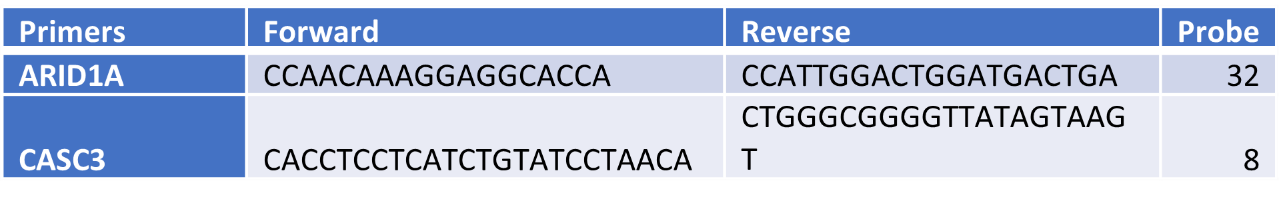


**Supplementary Table 1 Primers** QPCR primer target, forward and reverse sequences, and Roche Probe used for detection with Taqman Reagent.

**(A)**

**
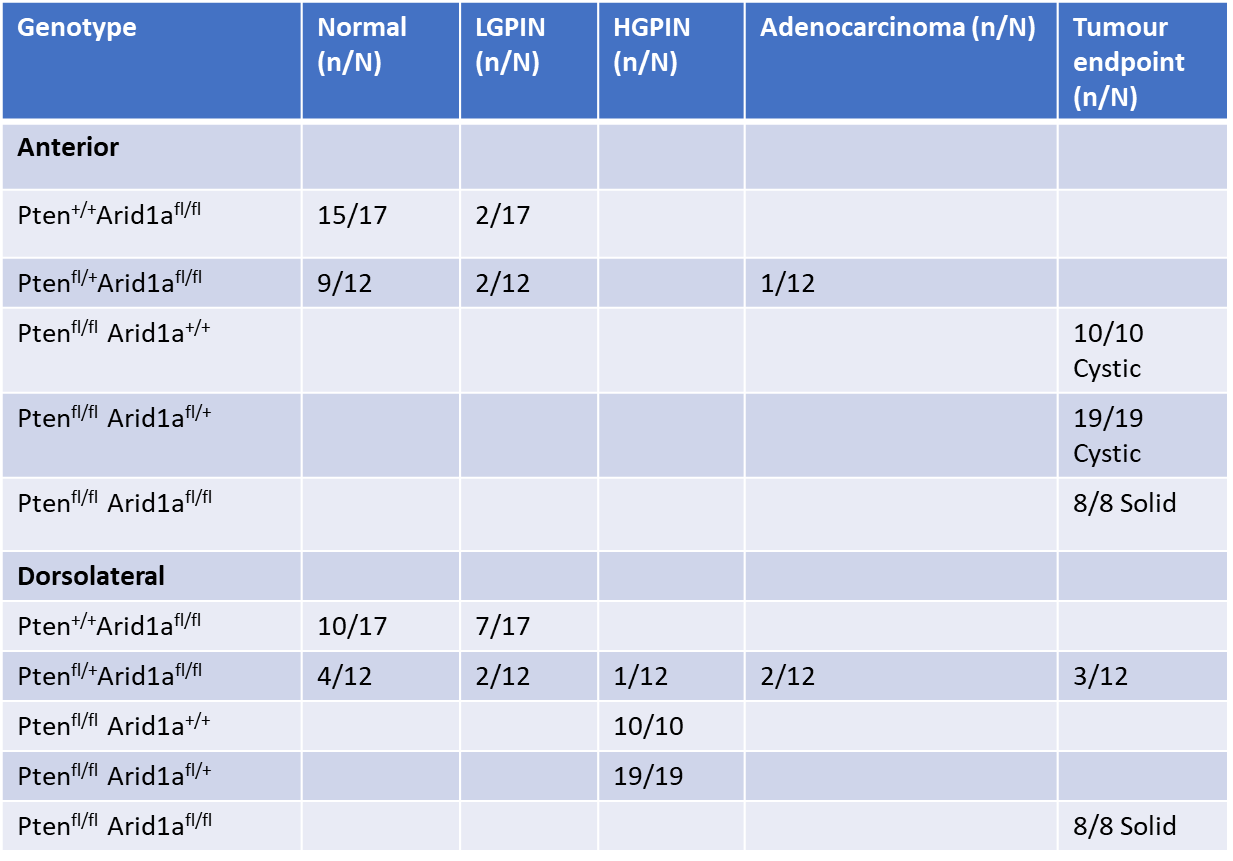
**

**(B)**

|  | ***Pten^fl/fl^ Arid1a^+/+^***  n/N | ***Pten^fl/fl^ Arid1a^fl/+^***  n/N | ***Pten^fl/fl^ Arid1a^fl/fl^***  n/N |
| --- | --- | --- | --- |
| **Anterior** | Cystic Tumours 10/10 | Cystic Tumours 19/19 | Solid Tumours 8/8 |
| **Dorsolateral** | No tumour | No tumour | Solid Tumours 8/8 |

**Supplementary Table 2.** (A) Proportions of mice which developed prostate cancer of anterior or dorsolateral prostate neoplasia as graded as resembling normal tissue, prostate intraepithelial neoplasm (either low grade prostate intraepithelial neoplasm (LGPIN) or high grade prostate intraepithelial neoplasm (HGPIN)), adenocarcinoma, and having reached clinical endpoint due to prostatic tumour**.** (n/N**,** number of mice affected/total number of mice in the group). (B) Extract of data from panel A to highlight the incidence of tumour formation in the anterior and dorsolateral lobes of the mice with homozygous *Pten* deletion and varying status of *Arid1a*. (n/N**,** number of mice affected/total number of mice in the group).


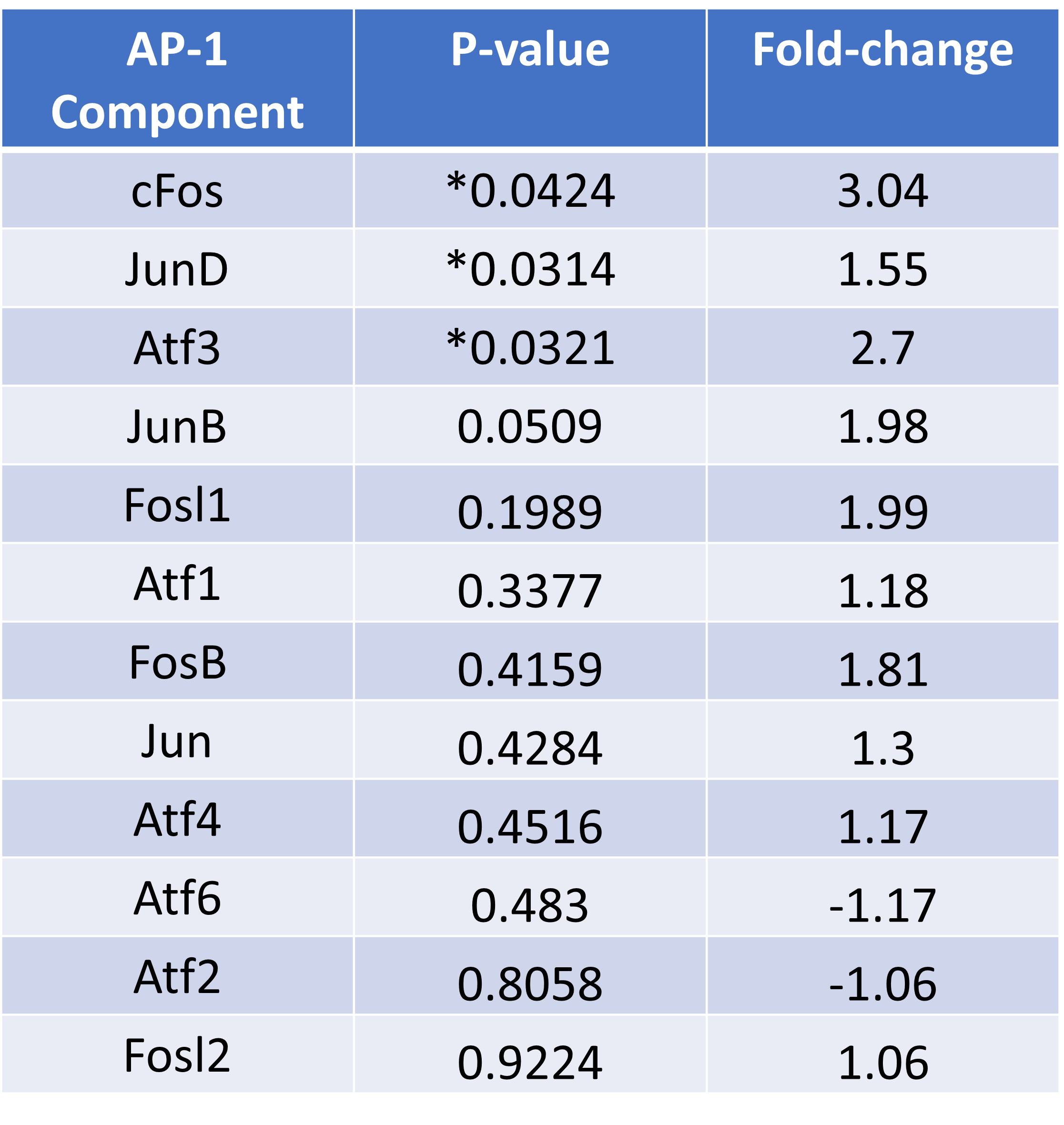


**Supplementary Table 3** AP-1 components showing fold change and degree of significance comparing *Pb-Cre;Pten^fl/fl^ Arid1a^fl/+^* (n=3) and *Pb-Cre;Pten^fl/fl^ Arid1a^fl/fl^* (n=3) cohorts.


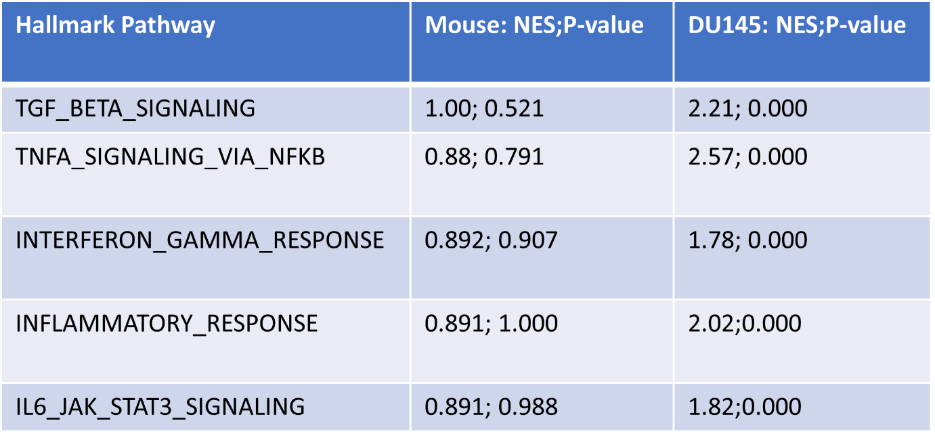


**Supplementary Table 4** GSEA performed comparing mouse cohorts *Pb-Cre;Pten^fl/fl^ Arid1a^fl/+^* (n=3) and *Pb-Cre;Pten^fl/fl^ Arid1a^fl/fl^* (n=3), and DU145 EV1 (n=5) vs ARID1A KO2 (n=5) cohorts. Indicated hallmark pathway shown along with normalised enrichment scores (NES), and P-values.
